# Sleep duration and the risk of new-onset arthritis in middle-aged and older adult population: results from prospective cohort study in China

**DOI:** 10.3389/fpubh.2024.1321860

**Published:** 2024-05-30

**Authors:** Qiangqiang Shang, Jie Zhou, Junjie Yao, Chaoqun Feng, Huijuan Lou, Deyu Cong

**Affiliations:** ^1^Department of Tuina, The First Affiliated Hospital of Changchun University of Chinese Medicine, Changchun, Jilin, China; ^2^Department of Anorectal, The First Affiliated Hospital of Changchun University of Chinese Medicine, Changchun, Jilin, China; ^3^College of Acupuncture and Tuina, Changchun University of Chinese Medicine, Changchun, Jilin, China; ^4^Hospital of Chengdu University of Traditional Chinese Medicine, Chengdu, Sichuan, China

**Keywords:** sleep duration, arthritis, new-onset arthritis, middle-aged and older adult, CHARLS, night sleep, daytime nap

## Abstract

**Background:**

The pain and sleep disorders caused by arthritis are health issues that have been re-emphasized with the aging population. However, the majority of research on arthritis and sleep disorders has focused on cases that have already been diagnosed with arthritis. This research aims to explore the correlation between sleep duration and new-onset arthritis in middle-aged and older adult individuals.

**Methods:**

Utilizing data from the China Health and Retirement Longitudinal Study from baseline (2011) to the Wave 3 follow-up (2018), we conducted a 7-year longitudinal investigation targeting populations with valid sleep questionnaire records and without arthritis. Sleep duration was assessed from nighttime sleep and daytime nap records. The new-onset of arthritis was determined based on self-reported diagnosis. We employed different logistic regression models to consider the potential impact of sleep duration on arthritis and conducted mediation analyses to assess the involvement of BMI in the association between sleep duration and the new-onset risk of arthritis.

**Results:**

Out of the 6,597 individuals analyzed in the cohort, 586 (8.9%) were diagnosed with new-onset arthritis. Median sleep duration was notably shorter in the new-onset arthritis group (6.63 vs. 6.41 h, *p* < 0.05). There was a notable negative correlation found between new-onset risk of arthritis and sleep duration, with each Interquartile Range (IQR) increment in sleep leading to a 16% risk reduction (OR: 0.864; 95% CI: 0.784–0.954). Stratified analyses revealed BMI as a potential modifier in the sleep-arthritis relationship (*P* for interaction = 0.05). Mediation analyses further showed that about 3.5% of the association was mediated by BMI. Additionally, the inclusion of sleep duration improved the arthritis predictive power of our model, with an IDI of 0.105 (0.0203, 0.1898) and an NRI of 0.0013 (0.0004, 0.0022) after adding sleep duration to the basic model.

**Conclusion:**

In the middle-aged and older adult demographic of China, increased sleep duration is associated with a decreased new-onset risk of arthritis, with BMI potentially playing a role in mediating this connection.

## 1 Introduction

With the continuous intensification of population aging and the increase in life expectancy, arthritis has gradually become one of the most burdensome musculoskeletal diseases (1, 2). Osteoarthritis (OA) and rheumatoid arthritis (RA) are the two types with the highest incidence rates, continuously affecting joints and synovial tissues, leading to pain, joint stiffness, limited mobility, and even joint deformities and functional loss for patients. This severely affects both their physical and mental health while also imposing enormous medical and economic burdens on individuals as well as society (3). In recent years, healthcare expenditure related to arthritis has been constantly increasing; however, studies have pointed out that this significant rise in healthcare expenditure has not improved the health-related quality of life for arthritis patients (4). Therefore, in this medical context, it may be a better choice to prevent the occurrence of arthritis by identify relevant risk factors and take early preventive measures.

The impact of arthritis on sleep has been extensively investigated. Pain and limited joint mobility, as the main clinical symptoms of arthritis, are closely associated with insomnia (5). Surveys report that up to 81% of arthritis patients experience insomnia. Additionally, a study from the US revealed that nearly one-third of arthritis patients reported various types of sleep disorders (6). Another from Korea found that almost 50% of OA patients experienced a significant reduction in sleep duration due to pain or vitamin D deficiency (7, 8). In summary, all sleep disturbances caused by arthritis directly lead to decreased sleep duration. On the other side of the coin, reduced sleep duration and changes in sleep structure are considered normal physiological phenomena during the aging process in humans; however, it has not been confirmed whether they have an impact on the occurrence of arthritis in middle-aged and older adult populations. Changes in hormone secretion related to sleep occur due to normal aging, which weakens the circadian rhythm regulation system and sleep stability of middle-aged and older adult individuals compared to younger people (9). For example, among those affected by normal aging, there is a significant decrease in sleep duration, efficiency, and slow wave sleep and a notable growth in wakefulness after sleep onset, awakening index, and percentage of time spent trying to fall asleep (10, 11). These changes in sleep patterns do not necessarily lead to the development of diseases as the body can still maintain a healthy physiological state by self-regulation. As individuals age, however, they may be more susceptible to developing primary sleep disorders like restless leg syndrome or sleep-disordered breathing. These increased disorders will present challenges to the restoration and maintenance of a stable sleep pattern, thereby disrupting the delicate balance of their sleep–wake cycle (12). Consequently, insufficient sleep among older adults puts them at risk for developing cardiovascular diseases, metabolic disorders, anxiety, depression, cognitive impairments and other conditions (11) that share similar lifestyle patterns and pathological features with arthritis patients (13, 14). Therefore, we hypothesize that the changed duration of sleep in middle-aged and older adult could significantly influence the occurrence of arthritis.

However, most of the research on risk factors related to arthritis has so far focused on diagnosed arthritis patients. For example, some studies have found a close association between cardiovascular diseases (15), poor quality living environment (16), and excessive use of toxic chemicals (17) with an increased risk of arthritis. By comparison, the exploration into the life trajectories of individuals newly diagnosed with arthritis is limited. Furthermore, large-scale population-based studies is lack for investigating sleep issues in newly developed arthritis patients. Therefore, we conducted a comprehensive analysis of the follow-up data on non-arthritic individuals from the China Health and Retirement Longitudinal Study (CHARLS) baseline population in 2011 to investigate the potential impact of sleep duration on the new-onset risk of arthritis in the general population, while also exploring potential associations with demographic characteristics.

## 2 Method

### 2.1 Study population

CHARLS conducted a longitudinal survey using stratified sampling and probability-proportional-to-size sampling methods, encompassing 450 communities/villages in 150 counties across China to track households and individuals aged 45 and above. Consequently, the CHARLS data exhibits high representativeness and accurately reflects the overall situation of the older adult population in both urban and rural regions across China. To date, CHARLS has released baseline data for 2011 as well as three follow-up survey datasets for 2013, 2015, and 2018. Approval for this study has been granted by the Ethics Committee at Peking University School of Medicine, with informed consent acquired from each participant prior to their involvement.

**Figure 1:**
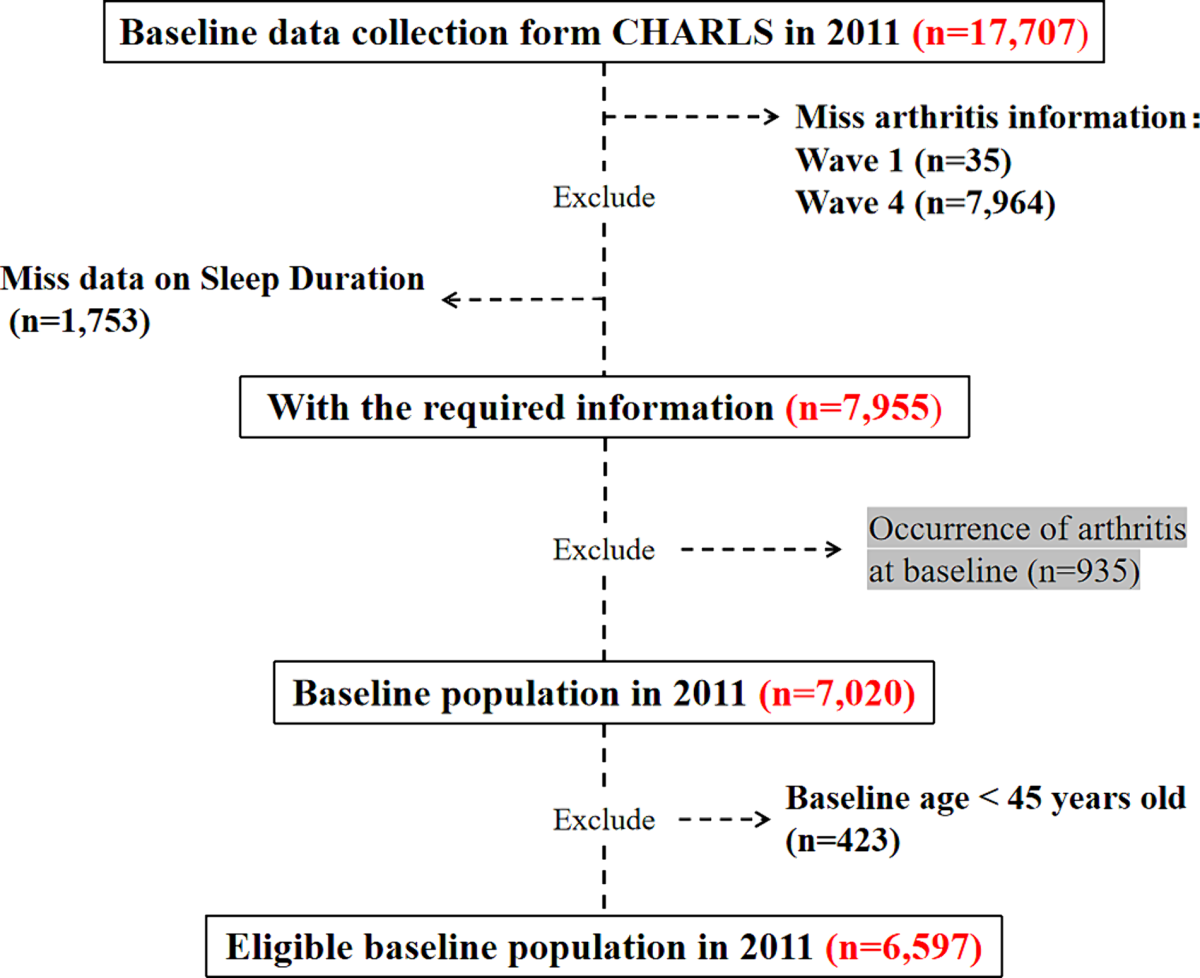
Flowchart of participant selection.

We conducted a 7-year longitudinal study (2011–2018), utilizing population data from 2011 as the baseline. The target population was chosen with valid records in the sleep questionnaire. After excluding participants diagnosed with arthritis at baseline or lacking arthritis information during the follow-up period, we longitudinally monitored and observed eligible participants until 2018. Among the 17,707 participants screened initially, 11,110 were excluded due to incomplete age information or age under 45 (*n* = 423), incomplete sleep duration or nap time information (1,753), incomplete information of arthritis at wave1 (*n* = 35) and wave 4 (*n* = 7,964), and confirmed diagnosis of arthritis at baseline (*n* = 925). Finally, the participants add up to 6,597 were included in the analysis (Figure 1), consisting of 6,011 individuals without arthritis and 586 individuals with new-onset arthritis. Figure 1 illustrates the flowchart depicting our study design.

### 2.2 Assessment of sleep duration

Sleep duration (in hours) is defined as the nighttime sleep reported by participants in their self-reported questionnaires; the specific time is determined based on the following question: “During the past month, how many hours of actual sleep did you get at night? “Daytime nap duration (in minutes) is determined based on the answer to the question: “During the past month, how long did you take a nap after lunch?”

### 2.3 Assessment of new-onset arthritis

The diagnosis of new-onset arthritis was based on self-reported data; when the interviewer asked, “Have you been diagnosed with arthritis by a doctor?” and the respondents answered “Yes,” they were classified as arthritis patients. Subjects who had arthritis in 2011 were excluded, and if the patient was diagnosed with arthritis after that until the follow-up period in 2018, he or she was included in the study under our definition of a patient with new-onset arthritis.

### 2.4 Assessment of covariates

Sociodemographic characteristics were included in this study, such as age, gender, marital status, education level (elementary school, below/high school or college and higher), and location (rural/urban). Lifestyle factors included smoking status and drinking status. Current diseases included hypertension and diabetes. In addition, different weight states, as defined by Body Mass Index (BMI) criteria, were also important covariates, including underweight (BMI < 18.5), health weight (18.5 ≤ BMI < 24), overweight (24 ≤ BMI < 28) and obesity (BMI ≥ 28), In our stratified analyses, we treated these as categorical variables, but for the logistic regression and mediation analyses, BMI was considered a continuous variable.

### 2.5 Statistical analysis

Continuous variables were articulated as mean ± standard deviation (SD) or medians (IQR), and categorical variables were gestured in percentages. Our research pivoted on the association between sleep duration, encompassing nighttime sleep and daytime nap duration as predictors, and the emergence of newly diagnosed arthritis as the outcome. We employed multivariate logistic regression analyses to delve into the association between sleep duration and new-onset risk of arthritis. Odds ratio (OR) with 95% confidence interval (CI) of sleep duration for new-onset risk of arthritis were calculated with three logistic models: Model 1, an unadjusted crude representation; Model 2, refined for sociodemographic variables such as age, gender, education, location, BMI, and marital status; and Model 3, further adjusted for confounders including smoking status, drinking status, hypertension, and diabetes. We initiated interaction analyses to explore potential moderating effect in the sleep-arthritis association due to variations in sociodemographic and health determinants. The dose–response relationship between sleep duration and new-onset risk of arthritis was elucidated visually using restricted cubic splines, demarcated at the 5th, 35th, 65th, and 95th percentiles. In assessing the auxiliary predictive prowess of sleep duration beyond conventional clinical markers, it was incorporated into a basic logistic regression model. For a holistic comparison, metrics such as the C-statistic, continuous Net Reclassification Improvement (NRI), and Integrated Discrimination Improvement (IDI) were utilized. The multiple interpolation method was used to impute the missing values. Data analysis Data analysis was performed with R 4.3.0.

## 3 Results

### 3.1 Study population characteristics

**Figure 2:**
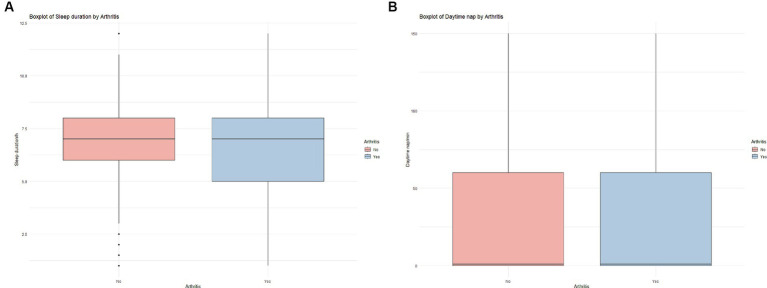
Box plots of the distribution of differences in sleep duration **(A)** and daytime nap **(B)**.

Table 1 delineates the characteristics of the study participants. The final cohort comprised 6,597 subjects, of which 586 were identified with newly diagnosed arthritis. The median age was 57 years, with males constituting 3,381 (51.25%) and females 3,216 (48.75%). A comparative analysis revealed that individuals with new-onset arthritis were predominantly female, had a lower educational attainment, and were non-smokers compared to their non-arthritic counterparts (*p* < 0.05). Notably, we discerned a shorter nocturnal sleep duration in new-onset arthritis patients (6.63 vs. 6.41 h, *p* < 0.05) (Figure 2).

**Table 1:** Baseline characteristics of study population by arthritis status at follow-up.

	Total (*n* = 6,597)	Non-Arthritis (*n* = 6,011)	Arthritis (*n* = 586)	*p*
**Age**	57 (50, 63)	57 (50, 63)	57 (50, 63)	0.87
**Gender**				<0.01
Female	3,216 (48.75)	2,892 (48.11)	324 (55.29)	
Male	3,381 (51.25)	3,119 (51.89)	262 (44.71)	
**Marital**				0.95
Married	5,977 (90.6)	5,447 (90.62)	530 (90.44)	
Non-Married	620 (9.4)	564 (9.38)	56 (9.56)	
**Education**				<0.01
Elementary school and below	3,979 (60.32)	3,592 (59.76)	387 (66.04)	
High school	1,623 (24.6)	1,485 (24.7)	138 (23.55)	
College and higher	995 (15.08)	934 (15.54)	61 (10.41)	
**Location**				0.13
Urban	5,861 (88.84)	5,329 (88.65)	532 (90.78)	
Rural	736 (11.16)	682 (11.35)	54 (9.22)	
**Smoking**				0.02
Never	3,908 (59.24)	3,532 (58.76)	376 (64.16)	
Former smoker	529 (8.02)	482 (8.02)	47 (8.02)	
Current smoker	2,160 (32.74)	1997 (33.22)	163 (27.82)	
**Drinking**				0.09
None of these	4,269 (64.71)	3,866 (64.32)	403 (68.77)	
Drink but less than once a month	546 (8.28)	505 (8.4)	41 (7)	
Drink more than once a month	1,782 (27.01)	1,640 (27.28)	142 (24.23)	
**Sleep duration**	7 (6, 8)	7 (6, 8)	7 (5, 8)	<0.01
**Daytime nap**	1 (0, 60)	1 (0, 60)	1 (0, 60)	0.86
**BMI**	23.33 (21.1, 25.68)	23.31 (21.08, 25.67)	23.61 (21.46, 25.83)	0.08
**Diabetes**				0.51
No	5,220 (79.13)	4,763 (79.24)	457 (77.99)	
Yes	1,377 (20.87)	1,248 (20.76)	129 (22.01)	
**Hypertension**				0.11
No	3,922 (59.45)	3,555 (59.14)	367 (62.63)	
Yes	2,675 (40.55)	2,456 (40.86)	219 (37.37)	

*p*-values were calculated from chi-square tests (categorical variables) or rank-sum tests (continuous variables without normal distribution), or *t*-tests (continuous variables with normal distribution). BMI, body mass index.

### 3.2 Longitudinal association between sleep duration and new-onset risk of arthritis

**Figure 3:**
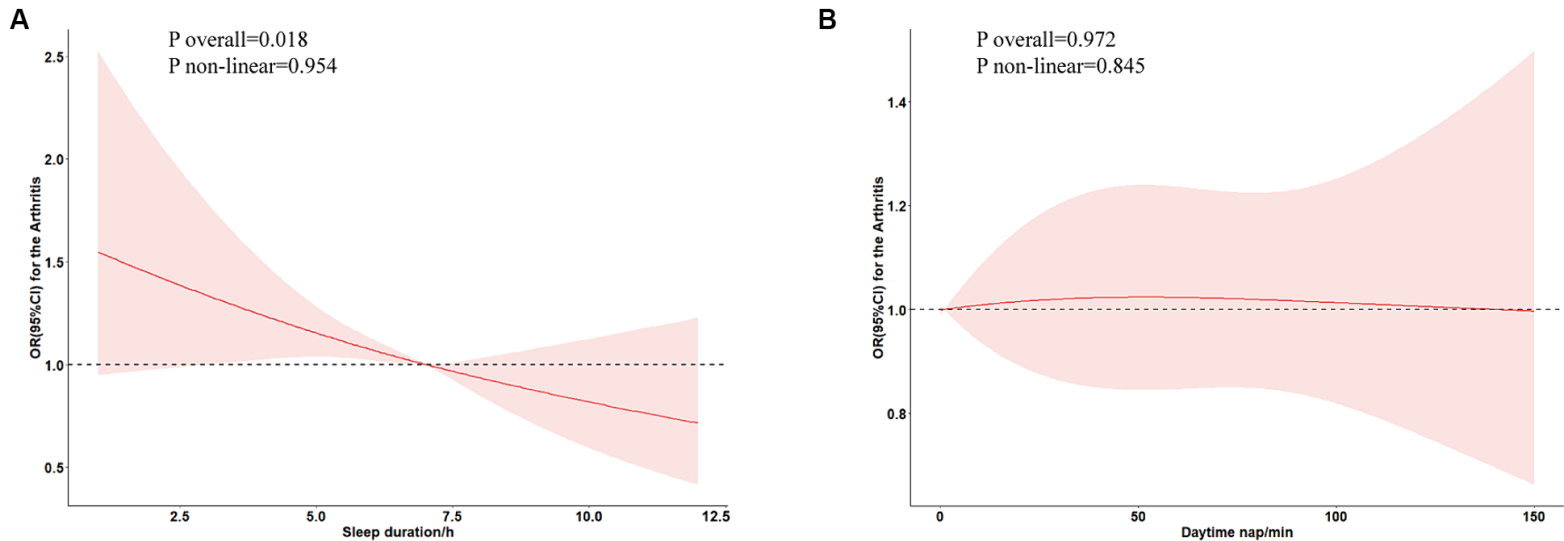
Association between sleep duration **(A)** and daytime nap **(B)** and new-onset risk of arthritis in restricted cubic spline analysis.

Table 2 summarizes the results derived from multivariate regression analyses. Evidently, when treated as a continuous variable, sleep duration demonstrated a significant inverse association with arthritis in a fully adjusted model. Specifically, there was a 16% risk reduction in new-onset arthritis for each IQR increment in sleep duration (OR: 0.864; 95% CI: 0.784–0.954). Upon stratifying sleep duration into categorical quartiles, the Q4 group, compared to the Q1 group, exhibited a pronounced 31% decline in arthritis onset (OR: 0.69; 95% CI: 0.48–0.96). However, no discernible correlation was observed between daytime nap and the new-onset risk of arthritis. Figure 3 shows a detailed insight into the Restricted Cubic Splines (RCS) evaluation, suggesting a linear dose–response relationship between sleep duration and new-onset risk of arthritis within the fully adjusted model (*P* overall = 0.018, *P* non-linear = 0.954). A critical threshold of approximately 6.91 h in sleep duration was identified as a pivotal determinant for the risk of arthritis onset, with the incidence showing an upward trend for sleep duration durations less than this threshold.

**Table 2:** Prospective associations between baseline sleep duration and daytime nap with follow-up new-onset arthritis in CHARLS.

Arthritis	Model 1	*p*	Model 2	*p*	Model 3	*p*
Sleep duration per IQR	0.861 (0.781, 0.95)	0.003	0.867 (0.786, 0.957)	0.004	0.864 (0.784, 0.954)	0.003
Q1	Ref		Ref		Ref	
Q2	0.79 [0.63, 0.99]	0.041	0.80 [0.64, 1.01]	0.06	0.80 [0.63, 1.00]	0.054
Q3	0.83 [0.67, 1.02]	0.078	0.83 [0.67, 1.03]	0.09	0.83 [0.67, 1.02]	0.083
Q4	0.72 [0.50, 0.99]	0.054	0.70 [0.49, 0.97]	0.038	0.69 [0.48, 0.96]	0.032
*P* for trend	0.016		0.014		0.012	
Daytime nap per IQR	0.999 (0.995,1.004)	0.770	1 (0.996,1.004)	0.955	1 (0.996,1.004)	0.944
Q1	Ref		Ref		Ref	
Q2	0.98 [0.63, 1.47]	0.931	1.00 [0.64, 1.50]	0.994	1.01 [0.64, 1.51]	0.981
Q3	1.00 [0.83, 1.21]	0.982	1.04 [0.86, 1.26]	0.651	1.05 [0.86, 1.27]	0.641
Q4	0.95 [0.73, 1.24]	0.725	0.99 [0.75, 1.29]	0.944	1.00 [0.76, 1.30]	0.983
*P* for trend	0.827		0.83		0.799	

Model 1 was crude model. Model 2 was adjusted for age, gender, education level, location, marital status and BMI. Model 3 was further for smoking status, drinking status, hypertension and diabetes.

### 3.3 Stratified analysis

**Figure 4:**
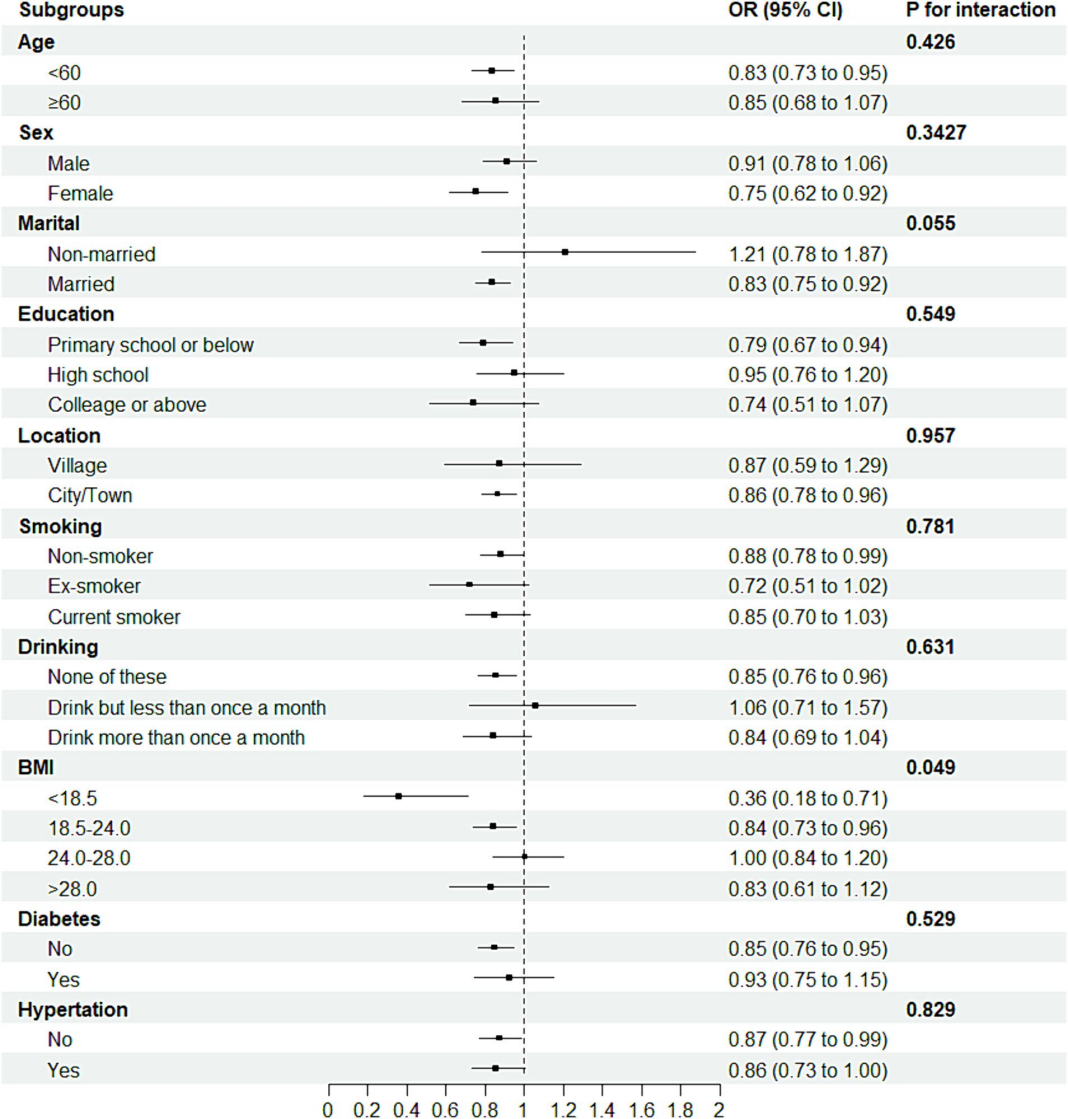
Forest plot of stratified analysis of the association of sleep duration and new-onset risk of arthritis.

To probe the robustness of the relationship between sleep duration and the new-onset risk of arthritis, we categorized participants into distinct subgroups based on sociodemographic variables and medical history, as delineated in Figure 4. Further interaction testing indicated a moderating effect of the BMI in the association between the sleep duration and the new-onset risk of arthritis (*P* for interaction = 0.05). Intriguingly, individuals with a BMI less than 24 seemed to benefit more from the impact of sleep duration on the arthritis. For example, for individuals with a BMI ranging from 18.5 to 24, sleep time for every additional IQR was associated with a 16.0% (95% CI: 3.9–26.6) less odds of arthritis, but there was no association for those with BMI higher than 24.

### 3.4 Mediation analyses

The mediation analyses indicate that the association between sleep duration and new-onset risk of arthritis was partially mediated by BMI, with indirect effect estimates of 0.00028 (95% CI: 0.00006, 0.000000) and − 0.00874 (95% CI: −0.01730, 0.000000). Specifically, this mediating effect intermediated by BMI accounts for 3.5% (indirect effect/total effect) (Table 3).

**Table 3:** Mediation of the association between sleep duration and new-onset arthritis in CHARLS by BMI.

Sleep duration – BMI – Arthritis		Estimate	95%CI Lower	95%CI Upper	*p*-value
	ACME (average)	0.00028	0.000058	0.000000	<0.001***
	ADE (average)	−0.00874	−0.017300	0.000000	<0.001 ***
	Prop. Mediated	−0.03540	−0.362000	−0.010000	<0.001 ***

CI, confidence interval; BMI, body mass index; ADE, average direct effect; ACME, average causal mediation effect.

### 3.5 Incremental predictive value of sleep duration

We tested whether sleep time improved the predictive power of the basic model (Table 4). We found that the C-statistic increased from 0.57 to 0.58 (*p* = 0.099) with an IDI of 0.105 (0.0203, 0.1898) and an NRI of 0.0013 (0.0004, 0.0022) after adding sleep duration to the basic model, suggesting that sleep duration may improve the predictive ability of arthritis.

**Table 4:** Incremental predictive value of sleep duration.

Arthritis	C Statistic (95% CI)	*p*-value	NRI (continuous) estimate (95% CI)	*p*-value	IDI estimate (95% CI)	*p*-value
Basic model	0.5702 (0.5464, 0.5941)		**Ref**		**Ref**	
Basic model + Sleep duration	0.5803 (0.5564, 0.6041)	0.0994	0.105 (0.0203, 0.1898)	0.01514	0.0013 (0.0004, 0.0022)	0.00635

The basic model included age, gender, education level, location, marital status, BMI, smoking status, drinking status. NRI, net reclassification improvement; IDI, integrated discrimination improvement.

## 4 Discussion

In this comprehensive investigation of the effect of sleep duration on the new-onset risk of arthritis, we conducted a 7-year prospective study involving 6,597 middle-aged and older adult individuals without any prior history of arthritis from the CHARLS. Among them, 586 individuals were identified as new-onset arthritis during the period of follow-up starting from 2011. The incidence rate of arthritis was higher among females, those with lower levels of education, and individuals with unhealthy lifestyle habits such as smoking and alcohol consumption. Importantly, a correlation was found between increased sleep duration and a lower likelihood of developing arthritis; for every one IQR increase in sleep duration, there was a corresponding 13.6% decrease in risk. This association remained consistent across different predictive models, highlighting the significance of considering sleep duration. No significant correlation was found between nap duration and new-onset risk of arthritis. Furthermore, BMI played a mediating role in this relationship. These findings underscore the importance of addressing sleep disturbances among middle-aged and older adult populations when contemplating preventive measures for arthritis.

Sleep is an active process of regulation, reorganization, and rejuvenation that plays a big part in the physiological functioning. Sleep disorders, such as shortened duration or disrupted patterns, have significant detrimental effects on human health, particularly contributing to the development of various chronic diseases (18). Arthritis represents one of the most burdensome musculoskeletal chronic conditions in an aging population. According to statistical data, the prevalence of arthritis among the older adult people in China is significantly high, with an overall incidence rate of 34.4% for individuals aged 45 and above (19). Our prospective cohort study has substantiated the association between reduced sleep duration and increased risk of arthritis, which aligns with previous cross-sectional studies and Mendelian research findings for both OA and RA (20–22). Among numerous regulatory mechanisms involved in sleep, inflammation response associated with sleep disorders, metabolic dysregulation, and neuroendocrine disruption within the immune system are considered pivotal mechanisms accelerating the onset of arthritis.

Diffuse low-grade inflammation may be a phenotypic feature of normal aging in the human body, and older individuals can actively adapt to and maintain a healthy physiological state during the aging process (23). Research has confirmed that insufficient sleep directly leads to excessive activation of inflammation, immune system dysfunction, and accelerated disruption of homeostasis during aging (24, 25). For instance, in middle-aged and older adult individuals experiencing passive sleep deprivation or a reduction in physiological sleep duration, there is an observed spontaneous production of interleukin (IL) and tumour necrosis factor (TNF) -α monocyte cytokines at elevated levels in peripheral blood (26, 27). Moreover, there is an increase in the spontaneous expression of signal transducer and activator of transcription 1 and 5 within the monocyte population (26). This indicates that insufficient sleep not only induces spontaneous cellular innate immunity but also collectively promotes the generation of an inflammatory microenvironment and accumulation of inflammatory factors. In patients with arthritis, a lack of sleep activates inflammatory signals such as IL-1α and IL-1β, which can inhibit serotonin synthesis and suppress the activity of wake-promoting neurons, promoting sleep. However, insufficient sleep can trigger joint pain related to arthritis and exacerbate pain responses, leading to an intensification of inflammatory reactions (24). Strong evidence from animal experiments also confirms the activating effect of sleep deprivation on inflammation. After acute sleep deprivation, mice showed a significant decrease in α and β diversity of gut microbiota, along with a significant increase in TNF-α levels. Overactivated inflammatory factors were transmitted to the brain through TNF receptor superfamily 1A, leading to significantly elevated levels of glial cell activation markers and chemokines in the cerebral cortex, thereby exacerbating systemic inflammation (28). On the other hand, chronic sleep deprivation resulted in a broader range of neuroinflammation and oxidative stress, including microglia and astrocyte activation, downregulation of acetylcholine receptors involved in the cholinergic anti-inflammatory pathway, as well as decreased downstream PI3K/AKT/GSK-3β activation. This was accompanied by an upregulation of pro-inflammatory cytokines and a downregulation of anti-inflammatory factors or antioxidant enzymes, such as Heme Oxygenase-1 levels (29). Therefore, sleep disorders can increase inflammation within the feedback loop maintained by downregulation of glucocorticoid receptor sensitivity and sympathetic nervous system arousal mechanisms. Of perhaps greater significance, however, is the fact that excessive accumulation of inflammation further worsens the occurrence of sleep disturbances. In addition, obstructive sleep apnea (OSA) is also one of the causes of sleep disorders and may have an inherent connection with the occurrence of arthritis. It is characterized by an escalating respiratory pause-hypopnea index and declining blood oxygen saturation with age, resulting in fragmented and shortened sleep duration. Extensive research has elucidated the intricate association between OSA and RA, with approximately half of RA patients experiencing varying degrees of OSA (30). The underlying mechanism may involve inadequate sleep and hypoxia induced by OSA, leading to oxidative/nitrosative stress, heightened inflammatory cytokines, imbalance in pro-inflammatory markers, impaired Nitric Oxide production, as well as endothelial dysfunction (31). For instance, Li et al.’s study on rats subjected to hypoxia treatment demonstrated enhanced expression of differentially expressed in chondrocytes and reduced expression of peroxisome proliferative activated receptor-γ directly contributing to elevated oxidative stress along with the expression of pro-inflammatory cytokines such as TNF-α, IL-1β and IL-6 (32).

Sleep is crucial for maintaining the neuroendocrine balance of the immune system. Insufficient sleep disrupts the hypothalamic–pituitary–adrenal (HPA) axis (33) and sympathetic nervous system (SNS) (34), increasing inflammatory responses and interfering with hormone levels. Correspondingly, this in turn may regulate neural adaptability and innate immune response (35). Research has shown that older individuals with sleep disorders not only exhibit increased expression of systemic inflammatory factors but also have elevated activity of transcription factors (TF) associated with immune activation and SNS function, while experiencing decreased activity of TF related to HPA axis function (27). This suggests a correlation between reduced sleep and gene expression patterns consistent with Conserved Transcriptional Response to Adversity activation. Furthermore, insufficient sleep in other healthy populations has been found to enhance inflammation expression, further inducing dysregulated HPA axis crosstalk during sleep–wake activity. This leads to excessive inflammation, increased sensitivity to pain (36, 37), as well as potential associations with heightened impulsivity, slower cognitive processing, and impaired executive functions (38). Temporary sleep deprivation enhances SNS activity, thereby modulating the release of inflammatory factors in both the bloodstream and brain. However, a study investigating insufficient sleep and decreased bone mass revealed that reduced leptin secretion resulting from sleep deficiency induces excessive SNS activation, leading to increased bone resorption and decreased cortical bone thickness (39), consequently elevating the risk of joint damage and arthritis development. When inflammation transitions from acute to chronic stages as observed in chronic sleep deprivation, it perpetuates a state of prolonged overexpression of inflammatory factors within the body. Furthermore, continuous activation of the SNS also exerts detrimental effects due to loss of sympathetic nerve fibers at inflamed sites during long-term inflammation, resulting in diminished norepinephrine levels. Consequently, local cells and immune cells acquire an endogenous ability to produce catecholamines for fine-tuning inflammation responses that have lost central control by the brain. However, if this process persists akin to chronic inflammation, sustained impairment in neural breakdown metabolism will expedite arthritis onset (40, 41).

Increased nocturnal metabolic rate serves as a compensatory mechanism to mitigate the negative consequences by poor sleep quality (42). Diminished sleep duration directly impacts various metabolic processes within the body and is significantly associated with an elevated risk of developing metabolic syndrome (43). Trails have demonstrated that sleep deprivation in healthy individuals leads to a substantial decline in insulin sensitivity (44), decreased secretion of appetite-regulating hormone leptin, heightened release of growth hormone-releasing peptide, and a noticeable increase in energy and fat intake due to enhanced food accessibility, ultimately culminating in weight gain or even obesity (45–47). Research conducted in multiple countries, including China and the United States, has shown that a high BMI index places significant strain on weight-bearing joints (48). The incidence of osteoarthritis in obese individuals may be 43% higher than in normal-weight individuals (49), establishing a clear causal relationship between genetically determined BMI and the susceptibility to arthritis development (50). This also elucidates why our study findings indicate that BMI acts as a pivotal moderating factor for both reduced sleep duration and increased new-onset risk of arthritis, corroborated by stratified analysis and mediation analysis. And stratified analysis also showed that being underweight (BMI < 18.5) may have the strongest regulatory effect, which indirectly explains why arthritis patients with lower body weight tend to respond better to specific treatments (51). In addition, reduced sleep duration also stimulates an increase in the secretion of various thyroid hormones (47). Thyroid hormones have been proven to exert significant effects on bone development and metabolism, particularly during adulthood. Elevated levels of thyroid hormones enhance the breakdown metabolism of joint bones (52, 53), which is closely associated with the early onset of arthritis. Insufficient and disrupted sleep also impacts solute transport in the brain, leading to impaired regulation of the HPA axis and a decreased metabolic rate. This manifests as sustained morning attention deficits and can also affect testosterone levels, decrease muscle mass, and limit functionality (54–56). Collectively, these detrimental effects may contribute to joint instability, increasing the risk of sports injuries and falls while damaging joints and synovial membranes, thereby accelerating arthritis occurrence.

Poor subjective sleep quality in the older adult may also contribute to the occurrence of arthritis at a psychological level. Research has demonstrated a significant correlation between long-term depressive symptoms and an increased risk of arthritis (57), as subjective psychological factors substantially amplify social psychological stress, which is closely associated with the development of chronic diseases. Furthermore, research on the relationship between arthritis and sleep patterns is notably scarce. ZHANG S et al. discovered that individuals who have longer nighttime sleep and shorter daytime sleep are less likely to develop arthritis compared to those who sleep less at night and longer during the day (58). However, our investigation did not reveal any noteworthy correlation between the daytime napping duration and the occurrence of arthritis. Sleep is a complex behaviour, especially napping, which can be influenced by culture and region. The impact of nap duration on health has shown different results; for example, in the study conducted by Jakubowski KP et al., napping was associated with increased inflammation risk (59). However, no definite conclusion could be drawn from an evaluation of a group of young individuals (60). Most researchers believe that napping cannot compensate for the negative effects of insufficient sleep duration on health and suggest extending nighttime sleep appropriately to ensure sufficient rest (61). We agree with this viewpoint and also consider changes in sleep patterns among older adults as one of the reasons (58).

As we know, this is the first instance where longitudinal cohort analysis has been employed to examine how sleep duration affects the new-onset risk of arthritis. It is worth noting that our research holds potential implications in identifying individuals at high risk and facilitating timely implementation of preventive measures, considering the significant burden associated with arthritis. Given that sleep disturbances-induced inflammatory response activation and neuroendocrine dysregulation are modifiable conditions, improving sleep quality may potentially reduce the new-onset risk of arthritis through various means such as maintaining a healthy BMI, avoiding trauma, and reducing psychological stress. However, stating certain inherent limitations in this study is necessary. Firstly, it is important to acknowledge that questionnaire data solely captures subjective sleep measurements and lacks objective sleep measurement methods. Nevertheless, it should be emphasized that subjective measurements play a vital role in the study of sleep. While self-reported sleep duration cannot entirely substitute objective measurements, it can still serve as an indicator for assessing variations in sleep quality and conducting large-scale population surveys. Secondly, due to the absence of comprehensive arthritis classification, we are unable to ascertain the precise impact of these findings on specific types of arthritis. Although a correlation has been established between sleep duration and overall arthritis, any conclusions drawn from this study regarding particular forms of arthritis should be interpreted with caution.

## 5 Conclusion

In this longitudinal cohort study, we have identified a correlation between sleep duration and new-onset risk of arthritis in the general population. Additionally, an elevated BMI may potentially mediate the association between sleep duration and the new-onset risk of arthritis. Therefore, we contend that it is imperative to underscore the significance of improving sleep quality and maintaining a reasonable weight among middle-aged and older adult individuals, which is pivotal for the primary prevention of arthritis.

## Data availability statement

Publicly available datasets were analyzed in this study. This data can be found at: http://charls.pku.edu.cn/pages/data/111/zh-cn.html.

## Ethics statement

The studies involving humans were approved by Ethics Committee at Peking University School of Medicine. The studies were conducted in accordance with the local legislation and institutional requirements. The participants provided their written informed consent to participate in this study.

## Author contributions

QS: Conceptualization, Methodology, Writing – original draft, Writing – review & editing. JZ: Formal analysis, Writing – original draft. JY: Conceptualization, Writing – original draft, Writing – review & editing. CF: Methodology, Software, Writing – original draft. HL: Conceptualization, Formal analysis, Writing – review & editing. DC: Funding acquisition, Investigation, Supervision, Writing – original draft, Writing – review & editing.
